# The Diagnostic Efficiency of Quantitative Diffusion Weighted Imaging in Differentiating Medulloblastoma from Posterior Fossa Tumors: A Systematic Review and Meta-Analysis

**DOI:** 10.3390/diagnostics12112796

**Published:** 2022-11-15

**Authors:** Yi Luo, Siqi Zhang, Weiting Tan, Guisen Lin, Yijiang Zhuang, Hongwu Zeng

**Affiliations:** 1 Shantou University Medical College, 22 Xinling Road, Jinping District, Shantou 515041, China; 2 Department of Radiology, Shenzhen Children’s Hospital, 7019 Yitian Road, Futian District, Shenzhen 518038, China; 3 Shenzhen Children’s Hospital of China Medical University, 7019 Yitian Road, Futian District, Shenzhen 518038, China

**Keywords:** posterior fossa tumor, diffusion-weighted imaging, medulloblastoma, meta-analysis

## Abstract

Medulloblastoma (MB) is considered the most common and highly malignant posterior fossa tumor (PFT) in children. The accurate preoperative diagnosis of MB is beneficial in choosing the appropriate surgical methods and treatment strategies. Diffusion-weighted imaging (DWI) has improved the accuracy of differential diagnosis of posterior fossa tumors. Nonetheless, further studies are needed to confirm its value for clinical application. This study aimed to evaluate the performance of DWI in differentiating MB from other PFT. A literature search was conducted using databases PubMed, Embase, and Web of Science for studies reporting the diagnostic performance of DWI for PFT from January 2000 to January 2022. A bivariate random-effects model was employed to evaluate the pooled sensitivities and specificities. A univariable meta-regression analysis was used to assess relevant factors for heterogeneity, and subgroup analyses were performed. A total of 15 studies with 823 patients were eligible for data extraction. Overall pooled sensitivity and specificity of DWI were 0.94 (95% confident interval [CI]: 0.89–0.97) and 0.94 (95% CI: 0.90–0.96) respectively. The area under the curve (AUC) of DWI was 0.98 (95% CI: 0.96–0.99). Heterogeneity was found in the sensitivity (I^2^ = 62.59%) and the specificity (I^2^ = 35.94%). Magnetic field intensity, region of interest definition and DWI diagnostic parameters are the factors that affect the diagnostic performance of DWI. DWI has excellent diagnostic accuracy for differentiating MB from other PFT. Hence, it is necessary to set DWI as a routine examination sequence for posterior fossa tumors.

## 1. Introduction

Posterior fossa tumors (PFT) are the most common central nervous system tumors in children, accounting for around 60% of pediatric brain tumors [1]. The pathological characteristics, malignant degree, metastasis frequency, and prognosis vary greatly among different histological types of PFT [2]. Accurate preoperative diagnosis plays a crucial role for pediatric patients with PFT, as the most common tumors in this location and age group, such as pilocytic astrocytoma (PA) and medulloblastoma (MB), may determine the need for different surgical approaches with markedly different outcomes and disease progression [3]. Medulloblastoma (MB) is the most common malignant tumor of the posterior fossa, accounting for about 40% of PFT [4]. While the current treatment strategy for MB is surgery combined with postoperative chemoradiotherapy [5], a recent study has reported that the complete resection rate of metastatic MB after neoadjuvant chemotherapy was higher, and the neuropsychological prognosis of children with delayed surgical resection was better [6]. The new treatment direction also emphasizes the importance of the preoperative diagnosis of MB.

Neuroimaging plays an extremely important role in preoperative diagnosis and evaluation [7]. However, due to the atypical or similar imaging features of various pathological types of PFT on conventional magnetic resonance imaging (MRI), accurate preoperative diagnosis may be challenging, such as the differentiation between MB and ependymoma (EP) [8]. 

Diffusion-weighted imaging (DWI) provides more microscopic information on tumor tissue by detecting the diffusion of water molecules in biological tissues, and generates apparent diffusion coefficient (ADC) values to quantitatively assess [9]. The motion of water in the interstitium is the main contributor to increased ADC values [10]. Malignant lesions usually have a smaller ADC value than benign lesions as a result of the narrow cytoplasm of tumor cells and the limited diffusion of water molecules [11]. At present, DWI has been applied for cancer diagnosis [12], tumor grading [13], and treatment response assessment [14]. Unfortunately, there is a large overlap in ADC values between different grades and types of tumors, making it unreliable to diagnose individual brain tumors using DWI alone [15,16,17]. In contrast, more studies suggested DWI can be highly accurate in distinguishing between malignant and benign lesions as well as the different histological types of PFT [18,19,20,21]. Meanwhile, there are remarkable differences in the formulation of the parameters of DWI and the definition of areas of interest in these studies, which lack systematic summary and analysis.

Thus, a comprehensive and systematic review is valuable for analyzing the available data from numerous existing studies. In this article, a meta-analysis is performed to evaluate the role of DWI as a tool for discriminating MB from other PFT.

## 2. Materials and Methods

This meta-analysis was conducted following the Preferred Reporting Items for Systematic Reviews and Meta-Analyses–Diagnostic Test Accuracy (PRISMA) Statement. Since our study was a systematic review and meta-analysis, Institutional Review Board approval and written informed consent were not required.

### 2.1. Literature Search

We searched PubMed, Web of Science, and Embase databases for studies published from January 2000 to January 2022. The keywords below were applied to search for eligible records: (diffusion OR diffusion-weighted imaging OR DWI OR apparent diffusion coefficient OR ADC) AND posterior fossa tumors. A search of the lists of references from included studies was also performed.

### 2.2. Study Selection

Two qualified authors (Y. Luo and S. Zhang) independently screened and checked the articles retrieved respectively, according to the predefined inclusion and exclusion criteria. Firstly, the title and abstract of the study were reviewed. Then the full text of potentially eligible studies was scanned swiftly. Finally, the eligible studies were screened by reading the full text carefully. Eligibility criteria were set as followings: (1) The tumors studied included MB; (2) DWI sequence and its related parameters were used for differential diagnosis; and (3) the purpose of the study was to explore the value of DWI in differentiating PFT and extracting true positive, false negative, false positive, and true negative results. Disagreement was resolved by discussion, and finally reaching a consensus.

### 2.3. Data Extraction and Quality Assessment

Data extraction was performed by author A (Y. Luo) and confirmed by author B (S. Zhang). The study characteristics extracted included country of origin, study type, reference standards, patients age, sample size, magnetic field strength, methods of ROI definition, *b* values (sec/mm^2^), sensitivity, specificity, the number of true positive, false negative, false positive, and true negative findings using DWI. For studies that reported multiple sensitivity and specificity in identifying posterior fossa tumors in different groups, we extracted the group with the highest number of correctly classified lesions (true-positive findings + true-negative findings) to avoid overrepresentation of a sample [22], whereas in subgroup analysis, we extracted each subgroup of data that we were interested in. Two investigators evaluated the risk of bias and applicability of each study independently by using the Quality Assessment of Diagnostic Accuracy Studies 2 (QUADAS-2) with any disagreement resolved with consensus.

### 2.4. Statistical Analysis

Meta-analysis was performed by using STATA version 16.0 (StataCorp, College Station, TX, USA). A bivariate random-effects model was used to calculate the pooled sensitivity and specificity. Heterogeneity of the pooled estimation was evaluated using the following criteria: (1) Cochran’s Q-test (*p* < 0.05 indicating the presence of heterogeneity); (2) Higgins inconsistency index (I^2^) test. Multilevel mixed-effects Logistic regression analysis was used to compare the diagnostic efficacy of DWI in differentiating MB from other PFT with a significant level of *p* < 0.05. Publication bias was assessed using Deeks’ funnel plots.

In order to explore the possible causes of heterogeneity, a meta-regression was conducted to evaluate the following factors: (1) the average age of the subjects (<18 years vs. >18 years); (2) country of origin (Asia vs. Non-Asia); (3) magnetic field strength (3.0 Tesla or mixed vs. 1.5 Tesla); (4) the calculation method of diagnostic parameters (only lesions are counted vs. normal tissue was referenced); (5) whether the optimal threshold value of diagnostic parameters is obtained from the ADC histogram; (6) the region of interest (ROI) in DWI or ADC map (single layer vs. multiple layers).

## 3. Results

### 3.1. Literature Search and Article Selection

**Figure 1:**
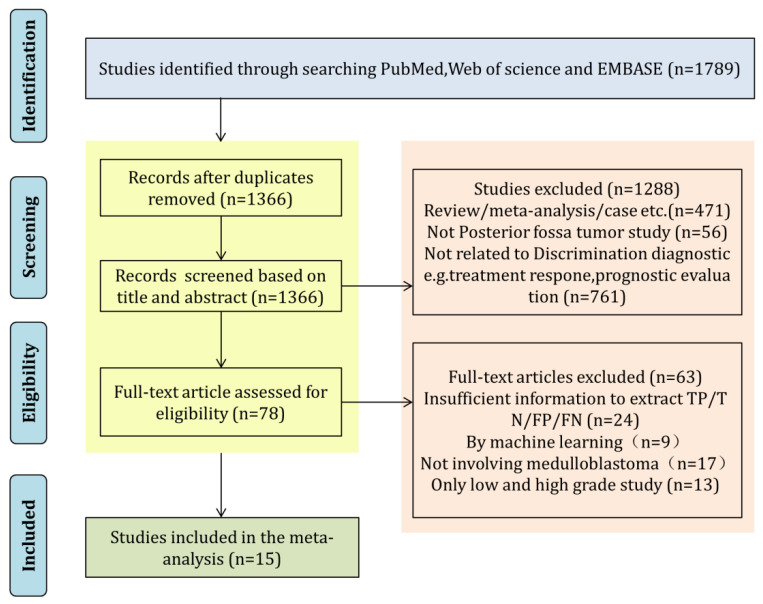
Flow chart describing the literature selection process.

A total of 1789 articles met the retrieval requirements (Figure 1), followed by the removal of 423 duplicate articles. After reviewing the titles and abstracts, 78 articles remained. Sixty-three articles were excluded by full-text review for the following reasons: 24 articles did not have enough data for calculation or construction of TP, FP, TN, and FN values; 9 articles used machine learning for analysis, 17 articles did not include MB, and 13 articles only identified high and low grade or benign and malignant tumors. Finally, a total of 15 studies was included for quantitative analysis [3,17,23,24,25,26,27,28,29,30,31,32,33,34,35].

### 3.2. Basic Characteristics of the Included Studies

The basic characteristics of the included studies were summarized in Table 1. The present meta-analysis included a total of 823 patients with PFT and 371 patients with MB among them. Six studies differentiated MB from EP, four studies differentiated MB from pilocytic astrocytoma (PA), and five studies differentiated MB from mixed PFT. Only two of the studies focused on adults, and the average age of patients in 12 of the studies was younger than 18. In one study the age of patients was not reported, we assumed that all patients were younger than 18 years old since the study sample was from a Children’s Hospital. Six studies obtained images with a magnetic field of 1.5 Tesla (T), seven studies used a combination of 1.5 T and 3.0 T, one study used 3.0 T, and one study did not report magnetic field. The majority of studies (14/15) were retrospective.

The diagnostic parameters of DWI and regions of interest selected by different studies were also heterogeneous. Minimum ADC value was used in two studies, mean ADC value was used in two, ADC ratio (the ratio of mean or minimum ADC value to normal tissue) was used in five, ADC histogram was used in five, and relative diffuse-weighted signal intensity (rDWSI) was used in one. While four studies took the entire tumor volume as their area of interest, four studies chose a single layer, and another seven studies chose three continuous or discontinuous layers. The reference standard for diagnosis in all studies was the pathological diagnosis. The DWI diagnostic parameters of all studies were measured without the pathological diagnosis.

### 3.3. Quality Assessment

**Figure 2:**
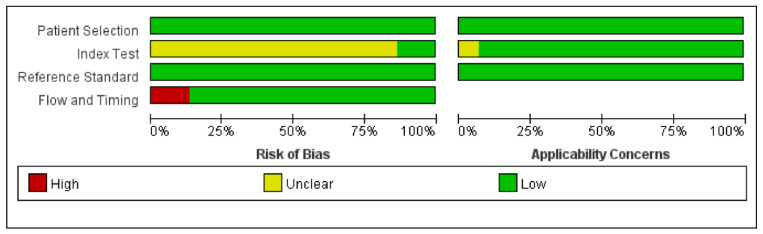
Results of quality assessment for risk of bias and concerns regarding the applicability of included studies. QUADAS-2 scores for each category are expressed by percentages of studies that have a low, high, or unclear risk of bias.

Figure 2 presents the results of quality assessment using QUADAS-2. The majority of the studies had high quality with a low risk of bias. All studies except two [30,32] did not report whether diagnostic thresholds were predetermined, so we marked them [3,17,23,24,25,26,27,28,29,31,33,34,35] as unclear risks of the index bias domain. Two studies [24,28] had a high risk of fluid and timing bias because the number of individual tumor types enrolled did not reach the basic level of statistical analysis. One study [30] had an unclear risk of concern of applicability for index test since the diagnostic index used in this study is unconventional and has not been reported in other studies, we doubt whether it can be replicated in clinical work. 

### 3.4. Main Statistical Analysis Results

**Figure 3:**
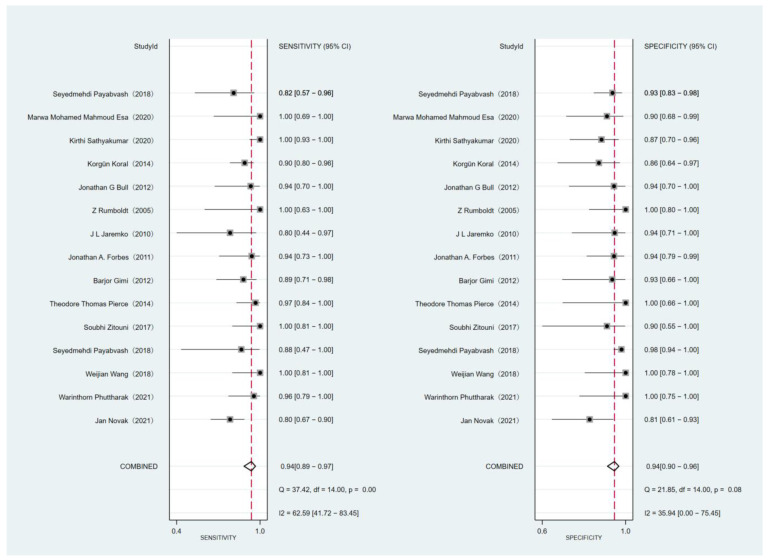
Forest plots of sensitivity and specificity with 95% confidence intervals per study.

**Figure 4:**
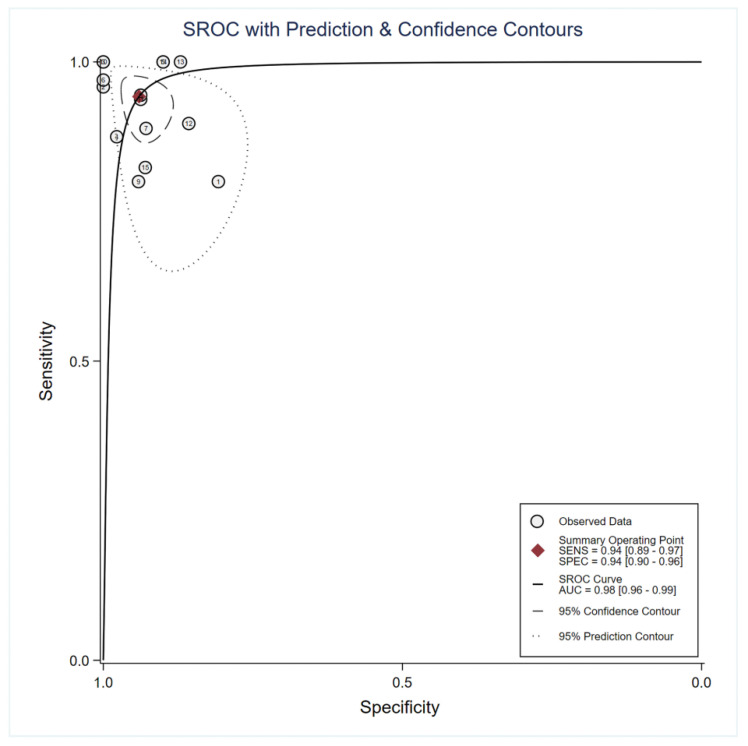
Summary receiver operating characteristic (SROC) curves of DWI.

**Figure 5:**
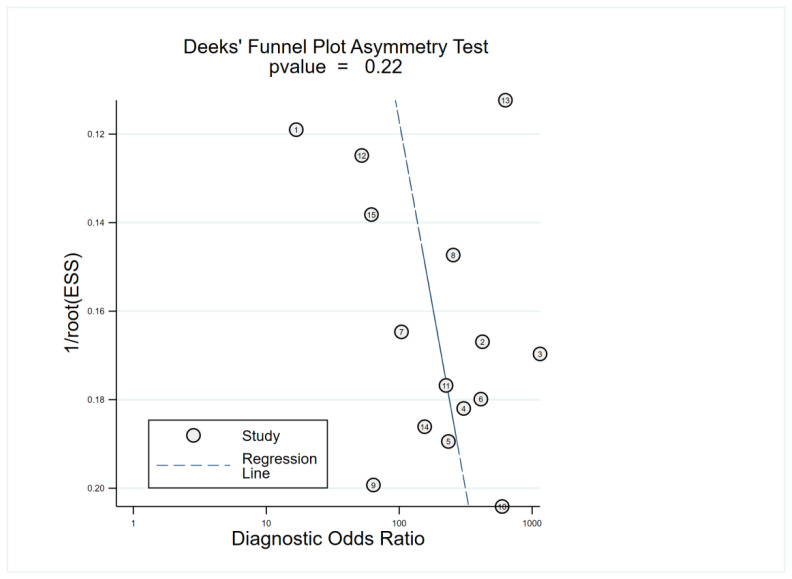
The funnel plot of publication bias for DWI.

The forest plot of the sensitivity and specificity of DWI is displayed in Figure 3. DWI showed a sensitivity of 0.94 (95% CI: 0.89–0.97), and a specificity of 0.94 (95% CI: 0.90–0.96) for differentiating MB from other posterior fossa tumors. The area under the curve (AUC) of ADC was 0.98 (95% CI: 0.96–0.99; Figure 4). The heterogeneity among studies was found for sensitivity (I^2^ = 62.59%) and specificity (I^2^ = 35.94) of DWI, but no threshold effect was identified (proportion of heterogeneity likely due to threshold effect = 0.80). Deeks’ funnel plot (Figure 5) shows there is no publication bias (*p* = 0.22). 

### 3.5. Subgroup Analysis and Meta-Regression

Among the covariates, magnetic field strength was revealed to be a significant factor affecting study heterogeneity (Table 2). Studies using 3.0 T scanners showed higher sensitivity of 0.98 (95% CI: 0.96–1.00) and specificity of 0.94 (95% CI: 0.91–0.98) than studies only using 1.5 T scanners which show the sensitivity of 0.89 (95% CI: 0.84–0.94) and specificity of 0.93 (95% CI: 0.87–0.98) (*p* < 0.05). The way the ROIs were delineated can also explain the heterogeneity of specificity, with the specificity of 0.93 (95% CI 0.89–0.96) for studies delineating a single layer and 0.97 (95% CI 0.93–1.00) for multiple layers (*p* = 0.01), which is not notably related to the heterogeneity of sensitivity (*p* = 0.13). Otherwise, the country of origin also explains the heterogeneity of sensitivity (*p* = 0.02), while the average age was not revealed to be a significant factor affecting study heterogeneity.

As for the differences in diagnostic parameters of DWI selected by different studies, our study conducted two grouping methods: one was directly obtained based on ADC images of lesions (min ADC, mean ADC, ADC histogram) and the other was calculated based on normal tissues (ADC ratio, rDWSI). Another grouping criterion was whether the diagnostic parameters were obtained directly from the PACS system, as the ADC histogram was calculated by other software. The results showed that the diagnostic parameters of DWI obtained by calculating the ratio of lesions to normal tissues have higher specificity of 0.95 (95% CI: 0.91–0.98) than those obtained based on lesions of 0.92 (95% CI: 0.87–0.98) with *p* < 0.001. However, whether DWI parameters were obtained directly from PACS had no remarkable effect on the efficacy of identifying PFT.

### 3.6. Comparison of DWI for Differentiating MB from EP and PA

**Figure 6:**
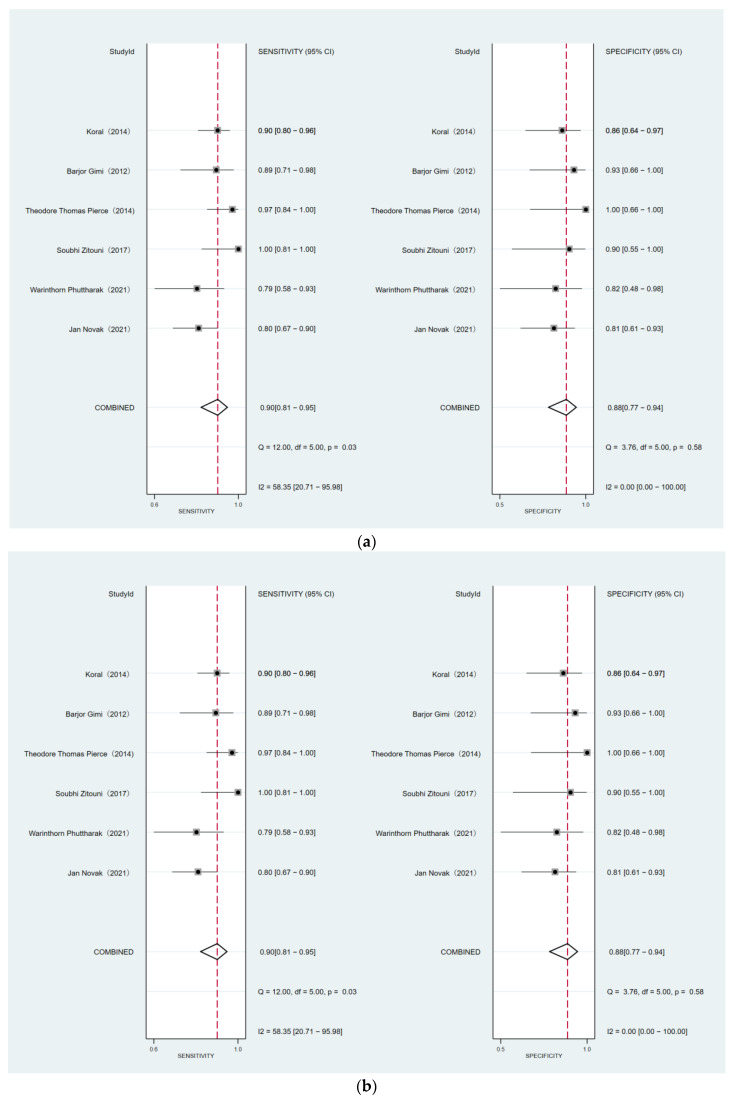
(**a**) The forest plot for differentiation MB between EP. (**b**) The forest plot for differentiation MB between PA.

A total of six studies were included that exhibit differentiation between MB and EP, and four studies that indicate the difference between MB and PA. The forest plots are shown in Figure 6. The sensitivity and specificity for DWI to differentiate MB from PA were 0.96 (95% CI: 0.77–1.00) and 0.99 (95% CI: 0.65–1.00), respectively. The sensitivity and specificity for DWI to differentiate MB from EP were 0.90 (95% CI: 0.81–0.95) and 0.88 (95% CI: 0.77–0.94), respectively (Table 3). There was no obvious difference in sensitivity (*p* = 0.74), while the difference in specificity (*p* = 0.01) was statistically significant. The key information of each study is shown in Table 4. The ADC ratio of MB was 0.91–1.02, and that of EP was 1.30–1.58. The optimal threshold value for differentiating MB from EP was 1.00–1.20.

## 4. Discussion

This meta-analysis investigated 15 studies with 823 patients to evaluate the diagnostic performance of DWI for differentiating MB from other posterior fossa tumors. DWI demonstrated an overall high diagnostic performance. The sensitivity of each study is heterogeneous, which is probably caused by magnetic field intensity, ROI determination method, and the diagnostic parameters of DWI. DWI showed the highest sensitivity and specificity in differentiating MB from PA. Therefore, we supposed that the quantitative DWI, as a non-invasive imaging inspection method, could improve the accuracy of the differential diagnosis for posterior fossa tumors. 

ADC value is the most regularly used diagnostic parameter of the DWI sequence, proved efficient in describing the limited diffusion of water molecules [36,37]. Compared with benign lesions, malignant lesions are characterized by more compressed cells, causing a reduction in water molecules, resulting in higher signal intensity but the ADC values decreased [38,39]. Fourteen of the fifteen studies selected derived parameters of the ADC map as diagnostic indicators. However, different studies have different calculation methods for ADC values. Previous studies have suggested that using the histogram to process DWI data may help to provide quantitative information on tumor heterogeneity and can be more advantageous in the differential diagnosis of different tumors and tumor grading [40,41,42]. Our results showed that the diagnostic performance of the ADC histogram was not significantly higher than that of other ADC parameters obtained directly from PACS system in differentiating posterior fossa tumors. It is more convenient and faster to use the ADC parameters directly obtained from the PACS system, such as ADC ratio, for radiologists in daily clinical practice. Moreover, the diagnostic specificity of DWI parameters can be enhanced by calculating the ratio of lesions to normal tissue (such as ADC ratio and rDWSI), which leads us to believe that the differences of individual normal tissues should be considered in the clinical application of DWI.

The definition of ROI used to calculate DWI diagnostic parameters was also of an important influence on the study. The ADC value of necrotic and cystic tumor components is very high compared to tumor tissue, so the inclusion of these areas would artificially increase the ADC value of the tumor [43]. Although the ROIs of our included studies did not select these components, it must be noted that the ROIs of the included studies still have differences. We found that the definition of ROI can account for the heterogeneity of specificity, with DWI parameters captured at multiple levels having higher specificity than those captured at a single level. Therefore, we recommend obtaining the DWI parameters by measuring the mean values of multiple layers rather than the mean values of different regions at the same layer.

In univariable meta-regression analysis, we also found that DWI showed better sensitivity and specificity in studies with high field intensity (3.0 T) MRI than in studies with only 1.5 T MRI. High field imaging can obtain a higher signal-to-noise ratio, thus improving the spatial resolution or signal-to-noise ratio of DWI images [44]. Our results suggested that high-field imaging may have better diagnostic performance in the differential diagnosis of posterior fossa tumors. This is consistent with many previous reports of DWI being applied to other organs or systems [36,45]. Since the 3.0 T has the advantages of improving signal-to-noise ratio and reducing artifacts such as medium effects, we suggest using 3.0 T DWI to improve the diagnostic performance of posterior fossa tumors. However, fewer than three studies only use 3.0 T MRI, and thus we cannot compare between 3.0 T and 1.5 T directly. In sequence, more subsequent studies will be needed to prove the validity of this comparison.

The three most common pediatric posterior fossa tumors were MB, EP, and PA [46]. There are important differences in the incidence, degree of malignancy, frequency of metastasis, and prognosis of these tumors, as well as the treatment strategies based on tumor type and histological subtype [47,48,49,50]. MB consists of densely arranged sheets of homogeneous small tumor cells with a small number of necrotic elements and normal tissue [10]. This histological feature of MB results in the lowest ADC value of the three tumors since the ADC value of brain tumors is related to the number of cells [51,52]. A previous meta-analysis also suggested that MB, EP, and PA had increased the mean ADC values sequentially [37]. Therefore, distinguishing MB from EP is more difficult than PA due to smaller differences in ADC values. This explains the statistically significant difference in the specificity of DWI for differentiating MB from EP than that for differentiating MB from PA. On the contrary, the results showed that the sensitivity of DWI for differentiating MB from EP was not significantly different from that of differentiating MB from PA. This means that quantitative DWI can be a beneficial auxiliary diagnostic tool when it is difficult to distinguish between MB and EP in the clinic.

At the same time, MB needs to be differentiated from atypical teratoma rhabdoid tumors (ATRT). Only one of the earlier studies included in this work evaluated the efficacy of DWI in differentiating MB from ATRT, and hence subgroup analysis could not be performed. This study [23] showed that the sensitivity and specificity of DWI in differentiating MB from ATRT were 66.7% and 50%, respectively. More studies [33,53] showed no significant difference in ADC value between MB and ATRT. This might be due to the fact that both MB and ATRT are embryonic tumors with large nuclei and small cytoplasm, the ADC values of the two tumors are small and similar, and it is difficult to distinguish them by DWI alone [53]. The preoperative differential diagnosis of MB and ATRT needs to be explored by more new MRI techniques.

In recent years, new Artificial Intelligence (AI) methods have emerged for the analysis of brain tumor imaging in children. Although the use of AI tools in routine clinical practice has yet to be explored, it has shown great potential in the identification of common PFT in children [54]. It is believed that the preoperative diagnosis of highly embryonic tumors in the posterior fossa may be more accurate in the future. Meanwhile, MRI-based AI technology is also used to predict the survival rate of brain tumor patients, which can provide supplementary information for improving clinical decision-making tasks. Combined with quantitative features derived from DWI, it is of great significance for AI to predict the survival assessment of brain tumor patients [55].

There were a few limitations of the present study. First, the number of eligible studies was relatively limited, with potentially relevant studies but no useful data reported to calculate TP, FN, FP, and TN. Second, the included studies showed significant heterogeneity in pooled sensitivities, which may reduce the general applicability of the combined estimates. However, we identified the cause of the heterogeneity through univariate meta-regression and found some methods that may enhance the diagnostic efficiency according to the meta-regression results. Finally, most of the included studies were retrospective studies (14/15). Confounding factors and bias were less controlled for than in prospective studies. Therefore, further large-scale prospective studies for diagnosing posterior fossa tumors should be conducted to provide a valuable reference for clinical diagnosis.

## 5. Conclusions

In conclusion, the diagnostic parameters of the DWI imaging sequence have excellent diagnostic accuracy for differentiating MB from other posterior fossa tumors. Standardizing the definition of the area of interest and the calculation method of diagnostic parameters will assist clinicians to improve the accuracy of diagnosis in daily work. High-field MRI, multi-slice ROI, and DWI parameters calculated with reference to normal tissues may be beneficial factors. The results of this study have the potential to provide valuable information for the treatment planning of pediatric PFT, including the extent of tumor re-section and the implementation of adjuvant therapy. Meanwhile, further prospective studies with standardized scanning protocols and large samples are needed to accurately quantify the diagnostic threshold. Quantitative DWI combined with artificial intelligence technology may be the future direction of in-depth exploration of posterior fossa tumors.

## Figures and Tables

**Table 1:** The study and patients’ basic characteristics of the included studies.

First Author/Year	Country	No. of Patients(n)	Age of Patients(y)	MB(n)	Other PFT(n)	Study Design	MRI Field Strength (T)	*b* Values (sec/mm^2^)	Selection of ROI	Diagnostic Parameters
Warinthorn Phuttharak (2021) [23]	Thailand	37	7.94	24	13	retrospective	1.5T/3.0T	0, 1000	single layer	ADC ratio
Jan Novak (2021) [24]	UK	81	5.8	55	26	retrospective	1.5T/3.0T	0, 1000	whole tumors	ADC histogram
Weijian Wang (2018) [25]	China	33	3–15	18	15	retrospective	3.0T	0, 1000	single layer	ADC histogram
Seyedmehdi Paybvash (2018) [26]	USA	142	47	8	134	retrospective	1.5T/3.0T	0, 1000	whole tumors	ADC histogram
Soubhi Zitouni (2017) [27]	Turkey	28	7.76	18	10	retrospective	1.5T	0, 1000	multiple layers	ADC ratio
Theodore Thomas Pierce (2014) [28]	USA	42	6.5	33	9	retrospective	NA	NA	multiple layers	ADC min
Barjor Gimi (2012) [29]	USA	41	5.9	27	14	retrospective	1.5T/3.0T	0, 1000	multiple layers	ADC ratio
Jonathan A. Forbes (2011) [30]	USA	50	<18	18	32	retrospective	1.5T	NA	single layer	rDWI
J L Jaremko (2010) [17]	Australia	27	5.8	10	17	retrospective	1.5T/3.0T	0, 1000	single layer	ADC min
Z Rumboldt (2005) [3]	USA	25	9	8	17	retrospective	1.5T	0, 1000	multiple layers	ADC mean
Jonathan G Bull (2012) [31]	UK	32	6.1	16	16	retrospective	1.5T	0, 1000	whole tumors	ADC histogram
Korgün Koral (2014) [32]	USA	79	6.8	58	21	retrospective	1.5T/3.0T	0, 1000	multiple layers	ADC ratio
Kirthi Sathyakumar (2020) [33]	India	82	8.24	51	31	retrospective	1.5T	0, 1000	multiple layers	ADC mean
Seyedmehdi Payabvash (2018) [34]	USA	74	25.4	17	57	retrospective	1.5T/3.0T	0, 1000	whole tumors	ADC histogram
Marwa Mohamed Mahmoud Esa (2020) [35]	Egypt	30	8.7	10	20	prospective	1.5T	0, 1000	multiple layers	ADC ratio

ADC = Apparent diffusion coefficient; rDWI = Relative diffuse-weighted signal intensity; MB = Medulloblastoma; MRI = Magnetic resonance imaging; NA = Not available; No. = number; PFT = Posterior fossa tumors; ROI = Region of interest; T = Tesla.

**Table 2:** Subgroup analysis and meta-regression for differentiating posterior fossa tumors.

Covariates	Subgroups	No. of Study	Sensitivity (95% CI)	*p*	Specificity (95% CI)	*p*
Age	<18	13	0.95 (0.91–0.98)	0.25	0.94 (0.90–0.97)	0.63
	>18	2	0.91 (0.79–1.00)		0.96 (0.90–1.00)	
						
Region	Asia	4	0.99 (0.98–1.00)	**0.02**	0.94 (0.87–1.00)	0.16
	Non-Asia	11	0.90 (0.86–0.95)		0.94 (0.91–0.97)	
						
Magnetic field strength	1.5 T	6	0.89 (0.84–0.94)	**<0.001**	0.93 (0.87–0.98)	**0.03**
	3.0 T or mixed	8	0.98 (0.96–1.00)		0.94 (0.91–0.98)	
						
DWI parameters	only lesions	9	0.94 (0.89–0.99)	0.17	0.92 (0.87–0.98)	**<0.001**
	with normal tissue	6	0.95 (0.90–0.99)		0.95 (0.91–0.98)	
						
ADC histogram	ADC histogram	5	0.93 (0.85–1.00)	0.26	0.97 (0.94–0.99)	0.18
	Not-histogram	10	0.95 (0.91–0.99)		0.91 (0.87–0.96)	
						
ROI	single layer	4	0.93 (0.89–0.98)	0.13	0.93 (0.89–0.96)	**0.01**
	multiple layers	11	0.96 (0.90–1.00)		0.97 (0.93–1.00)	

ADC = Apparent diffusion coefficient; DWI = Diffusion-weighted imaging; No. = number; ROI = Region of interest; T = Tesla. The *p*-values in bold indicate that the covariates are statistically significant.

**Table 3:** Differentiation of medulloblastoma from ependymoma and pilocytic astrocytoma.

No.of Studies	No.of Patients	Sensitivity	Heterogeneity		*p*	Specificity	Heterogeneity		*p*
			*p* Value	I^2^(%)			*p* Value	I^2^(%)	
Medulloblastoma vs. ependymoma									
6	316	0.90 (0.81, 0.95)	0.03	58.35	0.74	0.88 (0.77, 0.94)	0.58	0.00	**0.01**
Medulloblastoma vs. pilocytic astrocytoma									
4	122	0.96 (0.77, 1.00)	0.03	67.38		0.99 (0.65, 1.00)	0.14	45.18	

**Table 4:** (a) The information about studies that differentiate MB from EP. (b) The information about studies that differentiate MB from PA.

First Author/Year	MRI Field Strength (T)	Selection of ROI	Diagnostic Parameters	MB	EP	The Optimal Threshold Value
Warinthorn Phuttharak (2021)	1.5T/3.0T	single layer	ADC ratio	0.91 ± 0.17	1.3 ± 0.35	1.00
Jan Novak (2021)	1.5T/3.0T	whole tumors	ADC histogram (mean)	(0.87 ± 0.15) × 10^−3^ mm^2^ s^−1^	(1.13 ± 0.16) × 10^−3^ mm^2^ s^−1^	0.98 × 10^−3^ mm^2^ s^−1^
Soubhi Zitouni (2017)	1.5T	multiple layers	ADC ratio	1.02 ± 0.30	1.50 ± 0.20	1.18
Theodore Thomas Pierce (2014)	NA	multiple layers	ADC min	(0.54 ± 0.09) × 10^−3^ mm^2^ s^−1^	(0.88 ± 0.13) × 10^−3^ mm^2^ s^−1^	0.68 × 10^−3^ mm^2^ s^−1^
Barjor Gimi (2012)	1.5T/3.0T	multiple layers	ADC ratio	0.97–0.99	1.54–1.58	1.20
Korgün Koral (2014)	1.5T/3.0T	multiple layers	ADC ratio	——	——	1.20
Warinthorn Phuttharak (2021)	1.5T/3.0T	single layer	ADC ratio	0.91 ± 0.17	2.11 ± 0.51	1.17
Weijian Wang (2018)	3.0T	single layer	ADC histogram (mean)	106.5 ± 15.8	200.9 ± 31.4	137.7
J L Jaremko (2010)	1.5T/3.0T	single layer	ADC min	——	——	0.8 × 10^−3^ mm^2^ s^−1^
Z Rumboldt (2005)	1.5T	multiple layer	ADC mean	(1.65 ± 0.27) × 10^−3^ mm^2^ s^−1^	(0.66 ± 0.15) × 10^−3^ mm^2^ s^−1^	——

## Data Availability

The original contributions presented in the study are included in the article. Further inquiries may be directed to the corresponding author.
